# FEAR OF FALLING, FALL RISK, AND ETHNICITY AMONG OLDER ADULTS IN SINGAPORE: AN ELECTRONIC SURVEY

**DOI:** 10.1093/geroni/igac059.423

**Published:** 2022-12-20

**Authors:** Wayne Chong, Tharshini Lokanathan, W Quin Yow

**Affiliations:** Nanyang Technological University, Singapore, Singapore, Singapore; Singapore University of Technology & Design, Singapore, Singapore; Singapore University of Technology & Design, Singapore, Singapore

## Abstract

This study investigated the association between fear of falling and falls risk among Chinese and non-Chinese community-dwelling older adults in Singapore. Preliminary data were collected from individuals who will turn 60 years old and above in 2022 via an electronic survey between January and February 2022. The majority (61%) of the 128 Chinese participants had no falls risk and low fear of falling as compared to other combinations of falls risk (absence, presence) and fear of falling (low, moderate, high), Pearson Chi Square (2) = 8.03, p = .02. Among the 16 non-Chinese participants, no dominant falls risk-fear of falling combination was found, Pearson Chi Square (2) = 3.47, p = .18. Multinomial logistic regression analyses found, however, that in the presence of falls risk, ethnicity did not predict moderate fear of falling, B = .24, p = .70, nor high fear of falling, B = -.37, p = .74.

